# Social support predicted subsequent subjective well-being during the COVID-19 pandemic: a prospective study

**DOI:** 10.1186/s12889-024-18473-2

**Published:** 2024-04-02

**Authors:** Yanhui Mao, Junpeng Chen, Xinqi Liu, Junhua Dang, Helgi B. Schiöth

**Affiliations:** 1 Institute of Applied Psychology, Psychological Research and Counseling Center, Southwest Jiaotong University, Chengdu, China; 2 Department of Psychology and Behavioral Sciences, Zhejiang University, Hangzhou, China; 3 School of Social and Behavioral Sciences, Nanjing University, Nanjing, China; 4 School of Education, Huaibei Normal University, Anhui, China; 5 Anhui Engineering Research Center for Intelligent Computing and Application on Cognitive Behavior (ICACB), Anhui, China; 6 Department of Surgical Sciences, Uppsala University, Uppsala, Sweden

**Keywords:** Social support, Affect balance, Life satisfaction, Subjective well-being

## Abstract

**Background:**

Subjective well-being (SWB) is associated with social support in cross-sectional studies. However, it remains unclear whether and how social support predicts SWB longitudinally, especially during the COVID-19 contingency.

**Methods:**

By adopting a prospective design, the current work addressed this research question in a sample of 594 participants from the U.K. The data were collected via the online platform, Prolific, at two time points (June, 2020 and August, 2021) with a 14-month interval. Descriptive analysis and a moderated mediation model were conducted to test the proposed hypotheses.

**Results:**

Baseline social support was a significant predictor of subjective well-being (SWB) 14 months later, even after controlling for baseline SWB and other covariates such as personality traits. Additionally, affect balance (i.e., the affective component of SWB) fully mediated the link between baseline social support and subsequent life satisfaction (i.e., the cognitive component of SWB). Moreover, household income moderated this relationship, indicating a stronger mediation for individuals with lower monthly household income.

**Conclusion:**

The present work sheds light on the underlying mechanism and boundary condition of the association between social support and different components of SWB during the COVID-19 pandemic.

## Background

The COVID-19 pandemic has had a significant impact on people’s lives and mental health, leading to post-traumatic stress symptoms, confusion, financial loss, and increased rates of depression and anxiety disorders [1, 2]. To address these issues, recent studies have emphasized the importance of social support in mitigating the negative psychological effects caused by the quarantine [1], such as alleviated stress [3–5], lower loneliness [6–7], reduced anxiety [8, 9], and less depression [10]. The current paper focuses on the relationship between social support and subjective well-being (SWB) during the COVID-19 pandemic. Although studies have revealed a positive association between social support and SWB after the outbreak of the pandemic [11, 12], their limitations warrant attention. Firstly, these studies adopted cross-sectional designs, which provide very limited information for causal interpretations. Secondly, these works failed to consider the underlying mechanisms through which social support predicts SWB. Lastly, the boundary conditions of the relationship between social support and SWB have rarely been investigated.

Therefore, in this paper, we adopted a prospective design to investigate the predictive effect of social support on subsequent SWB during the COVID-19 pandemic. Specifically, in a sample of citizens living in the U.K., we tested the underlying mechanism of how perceived social support longitudinally predicted the cognitive component of SWB (i.e., life satisfaction) through the affective component of SWB (i.e., affect balance), after controlling for the baseline measure of SWB and other confounding factors such as personality traits. We also tested the boundary condition of when social support could predict future life satisfaction via affect balance by considering people’s household income.

### Social support

Social support has been studied enormously in past decades considering its significance in coping with disasters or crises [13]. Social support includes a variety of social interactions between friends, family members, neighbours, and others [14], and is usually defined as the existence or availability of those people on whom we can rely, and of those who let us know that they care about, value, and love us [15]. Social support is also believed to be supplied by the community, social networks, and confiding partners [16].

In terms of its conceptualization, social support can be defined by both a main effect model and a buffering effect model [17]. The main effect model conceptualizes social support as the extent to which a person is integrated in a large social network, whereas the buffering effect model conceptualizes social support as the availability of interpersonal resources that are responsive to the needs elicited by stressful events. Embeddedness in a social network is conducive to well-being because it precludes negative feelings resulting from social isolation and induces positive feelings of stability, predictability, and self-worth. However, the mere existence of a social network may not be necessarily beneficial in the face of stress. Instead, coping with stress requires the social network to provide relevant means and resources. Considering the stressful pandemic during which our study was conducted, we conceptualized social support based on the buffering effect model.

In general, the availability of interpersonal resources can be measured in two ways: One is the available assistance perceived by individuals, while the other is what they actually receive. It has been found that the former had greater influence on people’s mental well-being [18]. Similarly, compared with received social support, perceived social support also has a more substantial effect on various physical health outcomes such as cardiovascular disease and mortality [19]. Therefore, although social support can be gained from multiple sources and providers, what really matters is how people perceive the support they have received. In this paper, we aim to investigate how perceived social support is associated with different components of SWB.

### SWB

SWB encompasses both cognitive and affective aspects to measure an individual’s level of well-being [20]. The cognitive component of SWB, often referred to as life satisfaction, represents an individual’s overall evaluation of their life based on their personal values, priorities, and what the person deems important [21–23]. The affective component of SWB consists of both positive affect and negative affect. Positive affect includes a person’s desirable or pleasant emotions, such as enjoyment, gratitude, and contentment, whereas negative affect contains unwanted or unpleasant emotions, such as anger, sadness, and worry [24]. The coexistence of positive affect and negative affect is referred to as affect balance, which is distinct from but correlated with life satisfaction [25, 26].

Importantly, affect balance is often considered as an important information source of life satisfaction, with substantial studies reporting the mediation role played by affect balance in the relationship between various measures and life satisfaction [27, 28]. When people judge life satisfaction, they need to consider various aspects of their lives. According to the affect-as-information hypothesis [29], people typically rely on their affect balance (i.e., the extent to which they feel good or bad) to evaluate their life satisfaction (i.e., the extent to which they are satisfied with their lives). That is, affect balance is one of the most critical inputs of life satisfaction judgment. In line with this reasoning, it has been found that affect balance could mediate the effects of many predictors on life satisfaction, such as emotional intelligence [30, 31], self-esteem [32], social capital [33], and positive life attitudes [34]. However, these results were mainly based on cross-sectional studies. It remains unknown whether affect balance could mediate social support’s predictive effect on life satisfaction, especially in a prospective design.

### Social support and SWB

The idea that social support has a positive effect on health and well-being is widely accepted. When it comes to SWB, it has been consistently found that social support is associated with better affect balance and higher life satisfaction, both before the COVID-19 pandemic [30, 35], and during the pandemic [11]. However, the designs adopted in these studies are cross-sectional, which limits causal inferences. Therefore, in the current study, we aim to adopt a prospective design to test whether the baseline measure of social support could predict future affect balance and life satisfaction after controlling for the baseline measures of affect balance and life satisfaction. Considering that people often rely on their affect balance to evaluate their life satisfaction and that affect balance could mediate the effects of many predictors on life satisfaction, we will also test whether future affect balance mediates the relationship between baseline social support and future life satisfaction. We propose the following hypotheses.

#### Hypothesis 1

Baseline social support predicts subsequent affect balance.

#### Hypothesis 2

Baseline social support predicts subsequent life satisfaction.

#### Hypothesis 3

Subsequent affect balance mediates the relationship between baseline social support and subsequent life satisfaction.

Meanwhile, based on conservation of resources theory, the association between perceived social support and SWB might be moderated by household income. According to this theory, in order to protect themselves and cope with the challenges of daily life, individuals have to acquire and safeguard relevant resources, which include material resources such as money and properties, intrapersonal resources such as self-efficacy and growth mindsets, and interpersonal resources such as social support [36, 37]. Importantly, different types of resources can compensate for each other. For example, growth mindsets are particularly helpful in buffering against the deleterious effects of poverty on academic achievement [38, 39]. In our context, coping with stressful events such as the COVID-19 pandemic consumes resources, which in turn negatively affects well-being. However, such effect may vary depending on possessed material resources. Compared with rich people, those with low monthly household income tend to face more difficulties during the pandemic due to their lack of control in many domains of their lives [40–42], which makes them rely more on other types of resources such as social support. Therefore, we propose the following hypotheses.

#### Hypothesis 4

Household income moderates the mediating effect of affect balance in the relationship between social support and life satisfaction, such that the mediating effect is stronger for people with lower household income.

In order to rule out the confounding effects of demographic and personality factors, we control for age, gender, education, and the Big-Five personality traits when we test this proposed model (both the mediation and the moderated mediation).

## Methods

### Measures

#### Social support

The 12-item *Multidimensional Scale of Perceived Social Support* (MSPSS), developed by Zimet et al. [43], was applied in our study. It provides a measure of perceived support across three different dimensions (i.e., family, friends, and significant others), contributing to the understanding of an individual’s perceived availability of social support in their life, thus operationalizing functional support due to its focus on the functional aspects of support rather than the structural characteristics of social networks [14]. Sample items were “*My family really tried to help me”* and “*There is a special person who is around when I am in need*”. Responses for each item were ranked on a 7-point Likert scale from 1 (strongly disagree) to 7 (strongly agree). The internal consistency (Cronbach’s alpha) was 0.93.

#### Affect balance

We adopted the 12-item *Scale of Positive and Negative Experience* designed by Diener and colleagues to measure affect balance. This scale was designed to assess subjective feelings of positivity and negativity and has been shown to converge well with other measures of emotions [25]. This scale includes six items to assess positive affect (e.g., pleasant) and six items to assess negative affect (e.g., unpleasant). Respondents were asked to report how often they had experienced each of the twelve feelings measured in the scale over the past two weeks (“1” = “very rarely or never”, and “5” = “very often or always”). Cronbach’s alpha was 0.93 for positive affect at T_1_, 0.90 for negative affect at T_1_, 0.95 for positive affect at T_2_, and 0.92 for negative affect at T_2_ in the present dataset. Affect balance was obtained by subtracting negative affect from positive affect.

#### Life satisfaction

The 5-item *Satisfaction with Life Scale* [21], which was designed by Diener and colleagues to measure global cognitive judgments of satisfaction with one’s life, was adopted in the present work. Participants were asked to indicate their agreement with each of the five statements (e.g., “*In most ways, my life is close to my ideal*”). Responses were anchored on a 7-point Likert scale ranging from 1 (strongly disagree) to 7 (strongly agree), with higher scores indicating better satisfaction. Cronbach’s alpha was 0.91 and 0.93 for T_1_ and T_2_, respectively.

#### Demographics and personality

We measured gender (1 = Male, 2 = Female), age, educational level (1 = Primary school or less, 2 = Lower secondary school, 3 = Upper secondary school; 4 = Junior college, 5 = Bachelor, 6 = Master, 7 = Doctorate), and monthly household income (1 = £1,000 or less, 2 = £1,000 - £2,000, 3 = £2,000 - £3,000, 4 = £3,000 - £4,000, 5 = £4,000 - £5,000, 6 = £5,000 - £6,000, 7 = £6,000 - £7,000, 8 = £7,000 - £8,000, 9 = £8,000 - £9,000, 10 = £9,000 - £10,000, 11 = £10,000 or more). The Big-Five personality dimensions were measured by the Ten-item Personality Inventory (TIPI), a brief self-report questionnaire used to assess Big-Five personality traits: extraversion, agreeableness, conscientiousness, emotional stability, and openness to experience [44]. Each personality was measured by two adjectives. Sample items were “*I see myself as extraverted and/or enthusiastic.*” and “*I see myself as critical, and/or quarrelsome.*”. Participants rated themselves on a scale ranging from 1 to 7, indicating their agreement with each statement. It should be noted that the TIPI is designed to provide a quick assessment of personality traits and is often used in research studies where a more comprehensive measure of personality is not feasible or necessary.

### Participants and procedures

U.K. residents were recruited on an online platform (https://www.prolific.co/) for two sessions. In June 2020, 813 participants completed the first session (T_1_). They fulfilled the measures of affect balance, life satisfaction, social support, personality traits, and demographic factors, including age, gender, education, and household income. They were paid with £2. In August 2021, 594 participants completed the second session (T_2_), in which they fulfilled the measures of affect balance and life satisfaction again. They were paid with £1. Informed consent was obtained from all participants. Compared to participants who completed only the first session (lost group), those who completed both sessions (remaining group) were older (*M*_lost_group_ = 32.86, *M*_remaining_group_ = 42.45, *t* = 9.31, *p* < 0.001), included more females (*M*_lost_group_ = 1.53, *M*_remaining_group_ = 1.65, *t* = 3.07, *p* = 0.002), and had lower household income (*M*_lost_group_ = 4.85, *M*_remaining_group_ = 3.86, *t* = 4.60, *p* < 0.001). However, they had comparable education level (*M*_lost_group_ = 4.60, *M*_remaining group_ = 4.46, *t* = 1.62, *p* = 0.106). The details are shown in Table 1. We also did Little’s test to check the data, which showed that the data was not missing completely at random (MCAR), *χ*^2^ = 142.32, *p* < 0.001. Therefore, we replaced the missing values of each variable with the means of each variable, which yielded similar results as the main results we reported below.

Based on data from the 594 participants who finished both sessions, we tested whether social support measured at T_1_ would prospectively predict affect balance and life satisfaction measured at T_2_ after controlling for affect balance, life satisfaction, personality traits, and demographic factors measured at T_1_. The data are publicly accessible (https://osf.io/j2a8t/?view_only=6368555f2f494472bc77a2d841acc930).

**Table 1:** The differences in demographics between participants who completed both sessions and those who completed only the first session

Demographics		Participants completed T_1_ and T_2_ (*N* = 594)	Participants completed only T_1_ (*N* = 219)
Age	Mean (SD)	42.45 (13.74)	32.86 (10.93)
Gender	Male	207 (34.8%)	102 (46.6%)
	Female	387 (65.2%)	117 (53.4%)
Educational level	Primary school or less	0 (0.0%)	1 (0.5%)
	Lower secondary school	24 (4.0%)	2 (0.9%)
	Upper secondary school	114 (19.2%)	35 (16.0%)
	Junior college	117 (19.3%)	48 (21.9%)
	Bachelor	251 (42.3%)	95 (43.4%)
	Master	78 (13.1%)	35 (16.0%)
	Doctorate	10 (1.7%)	3 (1.4%)
Monthly household income	£1,000 or less	63 (10.6%)	15 (6.8%)
	£1,000 - £2,000	128 (21.5%)	28 (12.8%)
	£2,000 - £3,000	156 (26.3%)	57 (26.0%)
	£3,000 - £4,000	94 (15.8%)	41 (18.7%)
	£4,000 - £5,000	56 (9.4%)	14 (6.4%)
	£5,000 - £6,000	28 (4.7%)	13 (5.9%)
	£6,000 - £7,000	11 (1.9%)	7 (3.2%)
	£7,000 - £8,000	7 (1.2%)	8 (1.8%)
	£8,000 - £9,000	6 (1.0%)	3 (1.4%)
	£9,000 - £10,000,	3 (0.5%)	7 (3.2%)
	£10,000 or more	42 (7.1%)	30 (13.7%)

## Results

### Correlational analysis

Descriptive statistics of all measured variables and correlations among these variables are displayed in Tables 2 and 3, respectively. Results showed that social support measured at T_1_ was significantly and positively associated with affect balance and life satisfaction in both sessions. Affect balance measured at T_1_ had a high correlation with affect balance measured at T_2_. Life satisfaction showed a similar pattern. Therefore, it is essential to control for the baseline measures of affect balance and life satisfaction when estimating the longitudinal relationship between social support and future SWB.

**Table 2:** Descriptive statistics of all measured variables

Variable	Mean	SD	Skewness	Kurtosis
Age	42.45	13.74	0.34	-0.86
Gender	1.65	0.48	-0.64	-1.60
Educational level	4.46	1.11	-0.30	-0.49
Household income	3.86	2.57	1.56	1.93
Extraversion	3.36	1.53	0.41	-0.70
Agreeableness	4.93	1.18	-0.38	-0.03
Conscientiousness	5.32	1.20	-0.71	0.21
Emotional stability	4.38	1.45	-0.11	-0.79
Openness	4.55	1.18	-0.13	-0.45
Social support	61.50	15.32	-0.75	0.23
T_1_ Affect balance	4.20	9.14	-0.18	-0.34
T_1_ Life satisfaction	20.07	7.17	-0.29	-0.87
T_2_ Affect balance	5.19	9.90	-0.28	-0.41
T_2_ Life satisfaction	20.65	7.65	-0.32	-0.89

**Table 3:** Correlations among all measured variables

Variable	1	2	3	4	5	6	7	8	9	10	11	12	13
1 Age	-												
2 Gender	-0.08*	-											
3 Educational level	-0.14**	0.05	-										
4 Household income	-0.27	-0.02	0.03	-									
5 Extraversion	-0.29	0.12**	-0.03	0.06	-								
6 Agreeableness	0.21**	0.09*	-0.03	0.02	0.03	-							
7 Conscientiousness	0.13**	0.00	-0.01	0.06	0.11**	0.29**	-						
8 Emotional stability	0.18**	-0.14**	0.01	0.04	0.21**	0.37**	0.40**	-					
9 Openness	-0.05	-0.01	0.06	-0.03	0.28**	0.19**	0.17**	0.20**	-				
10 Social support	-0.00	0.06	-0.02	0.11**	0.29**	0.27**	0.34**	0.30**	0.15**	-	-		
11 T_1_ Affect balance	0.14**	-0.05	-0.00	0.11**	0.23**	0.31**	0.35**	0.61**	0.15**	0.47**	-		
12 T_1_ Life satisfaction	0.01	0.05	0.05	0.11*	0.20**	0.17**	0.28**	0.40**	0.11**	0.53**	0.68**	-	
13 T_2_ Affect balance	0.13**	0.03	-0.01	0.05	0.22**	0.32**	0.32**	0.51**	0.09*	0.45**	0.72**	0.56**	-
14 T_2_ Life satisfaction	0.03	0.07	0.08*	0.08	0.23**	0.20**	0.28**	0.37**	0.08	0.49**	0.61**	0.74**	0.76**

* *p* < 0.05, ** *p* < 0.01

**Table 4:** Regression results of the mediation

	T_2_ life satisfaction		T_2_ affect balance		T_2_ life satisfaction	
Predictor	β	*t*	β	*t*	β	*t*
Covariates						
Age	0.00	0.02	0.02	0.79	-0.01	-0.58
Gender	0.03	1.18	0.05	1.59	0.01	0.28
Education	0.06*	2.30	0.00	0.06	0.06**	2.84
Monthly household income	-0.02	-0.67	− 0.04	-1.43	0.01	0.24
Extraversion	0.07*	2.20	0.05	1.56	0.04	1.57
Agreeableness	0.02	0.73	0.08*	2.36	-0.02	-0.87
Conscientiousness	0.03	1.08	0.02	0.69	0.02	0.83
Emotion stability	0.01	0.16	0.09*	2.34	-0.05	-1.57
Openness	-0.06	-1.97	-0.06*	-2.06	-0.02	-0.91
T_1_ Affect balance	0.17**	3.91	0.54**	12.19	-0.15**	-3.87
T_1_ Life satisfaction	0.55**	13.92	0.09*	2.22	0.50**	15.70
Predictors						
T_1_ Social support	0.09*	2.58	0.09*	2.60	0.04	1.26
T_2_ Affect balance					0.59**	18.25
*R* ^2^	0.58		0.56		0.73	
*F*	67.10***		62.32**		122.98**	

* *p* < 0.05, ** *p* < 0.01. *β* is the standardized coefficient

### Mediating effect

We ran Model 4 of the PROCESS macro [45] plugged in SPSS to test whether T_2_ affect balance mediated the effect of T_1_ social support on T_2_ life satisfaction, with T_1_ affect balance, T_1_ life satisfaction, age, gender, education, and the Big-Five personality traits as covariates. First, the second column in Table 4 showed that T_1_ social support (i.e., the predictor) significantly predicted T_2_ life satisfaction (i.e., the outcome) after controlling for T_1_ affect balance, T_1_ life satisfaction, and other covariates (parameter *c* in the mediation analysis), *β* = 0.09, *p* =.011. Second, as shown in the fourth column in Table 4, T_1_ social support (i.e., the predictor) significantly predicted T_2_ affect balance (i.e., the mediator) after controlling for covariates (parameter *a* in the mediation analysis), *β* = 0.09, *p* =.011. Finally, as shown in the sixth column in Table 4, when T_1_ social support (i.e., the predictor) and T_2_ affect balance (i.e., the mediator) was simultaneously entered, T_1_ social support was no longer a significant predictor of T_2_ life satisfaction (parameter *c’* in the mediation analysis), *β* = 0.04, *p* = 0.203, whereas T_2_ affect balance (i.e., the mediator) was still significant (parameter *b* in the mediation analysis), *β* = 0.59, *p* < 0.001. The bootstrap estimation procedure with 5,000 bootstrapping samples showed that the total effect was 0.044. The indirect effect was 0.027 (61.36% of the total effect), SE = 0.010, 95%CI [0.007, 0.047] and the direct effect was 0.017, SE = 0.014, 95%CI [-0.010, 0.044], thus suggesting a full mediation. Note in all regressions, the variance inflation factor for each variable was between 1 and 3, thus showing there is no problem of multicollinearity.

### Moderated mediation

**Fig. 1:**
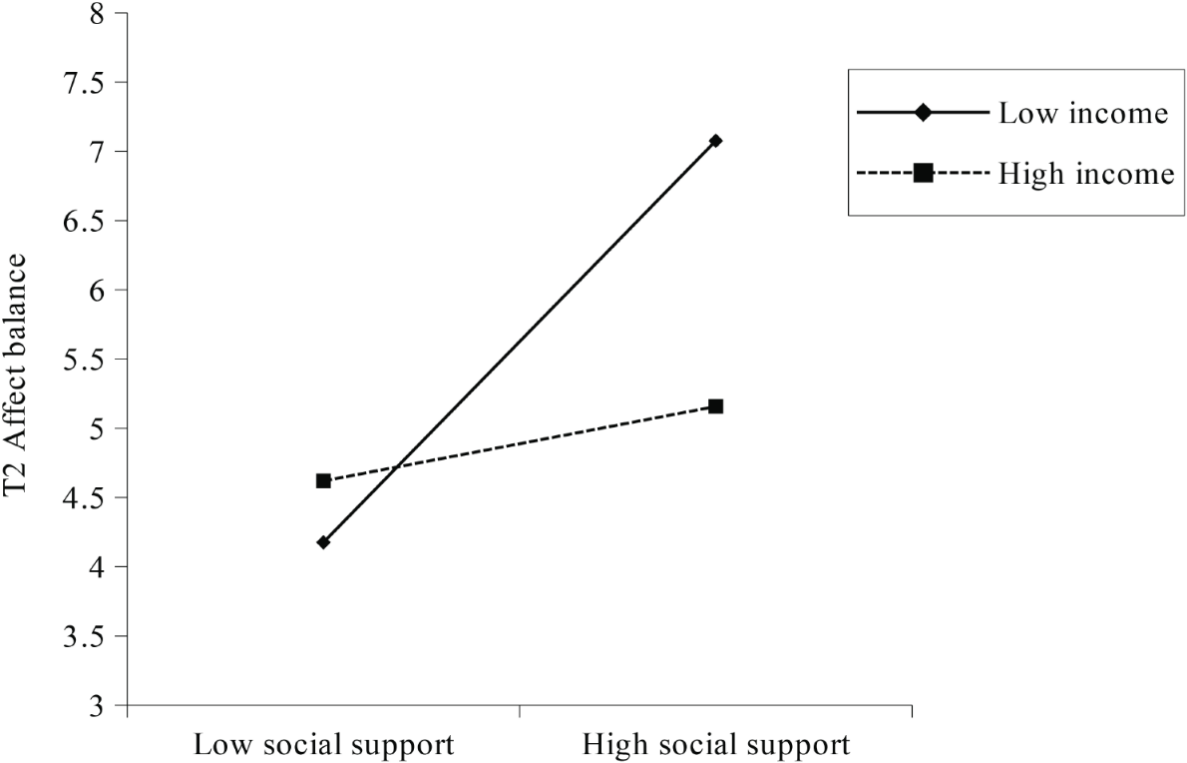
The moderating effect of monthly household income on the relationship between T_1_ social support and T_2_ affect balance after controlling for covariates

We ran Model 7 of the PROCESS macro to test whether monthly household income could moderate the relationship between T_1_ social support and T_2_ affect balance as well as the mediating effect of T_2_ affect balance on the relationship between T_1_ social support and T_2_ life satisfaction, with T_1_ affect balance, T_1_ life satisfaction, age, gender, education, and the Big-Five personality traits as covariates. As shown in Table 5, the interactional effect of social support and monthly household income on T_2_ affect balance was significant, *β* = -0.27, *p* = 0.029. Simple slope analysis showed that social support predicted T_2_ affect balance when monthly household income was low (1 SD below the mean), *β* = 0.09, *t* = 3.39, *p* < 0.001. However, when monthly household income was high (1 SD above the mean), social support no longer predicted T_2_ affect balance, *β* = 0.02, *t* = 0.53, *p* = 0.599. The pattern is depicted in Fig. 1.

**Table 5:** Regression results of the moderation

	T_2_ affect balance	
Predictor	β	*t*
Covariates		
Age	0.03	0.89
Gender	0.04	1.50
Education	0.00	0.03
Extraversion	0.05	1.53
Agreeableness	0.08*	2.53
Conscientiousness	0.02	0.62
Emotion stability	0.09*	2.34
Openness	-0.06	-1.90
T_1_ Affect balance	0.54**	12.28
T_1_ Life satisfaction	0.09*	2.15
Predictors		
Monthly household income	0.20	1.77
T_1_ Social support	0.17**	3.37
Social support × Income	-0.27*	-2.19
*R* ^2^	0.57	
*F*	58.27**	

* *p* < 0.05, ** *p* < 0.01. *β* is the standardized coefficient

The bootstrap estimation procedure with 5,000 bootstrapping samples revealed a significant moderated mediation, Effect = -0.007, SE = 0.003, 95%CI [-0.013, -0.001]. Specifically, the mediating effect of T_2_ affect balance on the relationship between social support and T_2_ life satisfaction was significant when monthly household income was low (1 SD below the mean), Effect = 0.043, SE = 0.013, 95%CI [0.018, 0.068], but not significant when monthly household income was high (1 SD above the mean), Effect = 0.007, SE = 0.014, 95%CI [-0.019, 0.035].

## Discussion

Prior studies testing the relationship between social support and SWB were mainly based on cross-sectional surveys. Although a few of studies employed a longitudinal design, they only considered social support and life satisfaction and did not separate SWB into its respective affective and cognitive dimensions [46, 47]. The current paper deepens the current understanding of this relationship by adopting a prospective design and investigating its underlying mechanism and boundary condition, particularly during the COVID-19 pandemic. We found that baseline social support could prospectively predict future life satisfaction via affect balance. This mediating effect was further moderated by household income, such that the mediation was stronger for people with lower monthly household income. Below we will discuss these results in a broader context.

First, results of the correlational analysis indicated that perceived social support was significantly correlated with affect balance and life satisfaction, both cross-sectionally and longitudinally. This is in accordance with and extends prior cross-sectional studies holding a positive association between social support and SWB [11, 30, 35]. Meanwhile, affect balance and life satisfaction measured at T_1_ were strongly correlated with affect balance and life satisfaction measured at T_2_, respectively, disclosing that it is necessary to control for the baseline measures of the two components of SWB while estimating the longitudinal relationship between social support and future SWB. Regarding the relationship between income and SWB, previous studies consistently found a positive association at a specific time point (i.e., cross-sectional design) [48]. Similarly, we also found significant correlations between monthly household income (measured at T_1_) and the two components of SWB at T_1_. However, monthly household income (measured at T_1_) was not correlated with the two components of SWB at T_2_. These findings align with a recent meta-analysis showing that the longitudinal association between objective socioeconomic status (i.e., income and education) and SWB was smaller than the cross-sectional association between them [48]. Considering the current special period during which the pandemic has dramatically influenced people’s lives, the contribution of income to prospective SWB may further decrease, as shown in our results.

Second, taking advantage of the prospective design and data entries collected at two different time points (T_1_ & T_2_), we tested whether there was a prospective association between social support and SWB (affect balance and life satisfaction). It was found that baseline (T_1_) social support significantly predicted future (T_2_) affect balance and life satisfaction, and T_2_ affect balance fully mediated the relationship between T_1_ social support and T_2_ life satisfaction, after controlling for the baseline measure of SWB and other confounding factors such as personality traits. This is a step forward for prior studies that only explored the mediation effect of affect balance on many other life satisfaction predictors (e.g., emotional intelligence, self-esteem) [30–33], but not precisely the link from social support to life satisfaction. This full mediation can be interpreted based on the affect-as-information hypothesis [29], which assumes that people make judgments and decisions concerning life satisfaction according to, for the most part, their own feelings [49]. Although judgments of life satisfaction may also be determined by other parameters in addition to affective feelings, they draw power from social support primarily through the pathway of affective feelings. This is also in line with the buffering effect model of social support, such that the social network needs to provide relevant means and resources to help the receivers of social support cope with the stress. In our situation, such means and resources increased the receivers’ positive feelings and alleviated their negative feelings during the pandemic, which in turns promoted their life satisfaction.

Finally, with regard to the boundary condition, results confirmed that the relationship between social support and affect balance was moderated by monthly household income. Notably, the effect of social support on affect balance was significantly stronger for U.K. citizens who reported lower monthly household income. This aligns with the coping strategies adopted by different social classes [50]. It is well documented that both social support and wealth can serve protective functions against the threat [51, 52]. People from the lower class are more likely to rely on others in the social environment because they have fewer material resources. By contrast, upper-class individuals tend to prioritize material wealth since wealth can afford them greater autonomy and self-reliance [50]. Therefore, during the current pandemic that poses a great threat to people’s social lives, social support could predict subsequent SWB for lower-income individuals because they rely on and value these communal resources to a larger extent. However, higher-income individuals may typically turn to material resources when coping with the pandemic. As a result, social support is not a critical determinant of their SWB.

### Limitations

This study has several limitations that can be considered and addressed in future work. First, findings based on the U.K. sample may not imply a fit-for-all solution for people worldwide. Therefore, replications of the findings in other countries or cultures may help to address the robustness and generalizability of our proposed model. Second, although our study overcame the shortcomings of the cross-sectional design by adopting a prospective design, its data were collected from only two waves. Future work applying longitudinal designs with more time intervals will provide more plausible inferences. Third, although we measured SWB twice, we measured social support only once at T_1_, which prevents us from testing whether there is a reciprocal relationship between social support and SWB with a more complex model such as the cross-lagged panel model. Finally, our findings are context-dependent, as it was conducted during the special period of the COVID-19 pandemic, a time when the world’s economy was severely hit and stagnated, which may make the moderating function of household income different from other periods.

## Conclusions

Prior studies have explored and proved the positive association between social support and SWB with cross-sectional evidence, but the causal inference behind it remains elusive. By adopting a prospective design, the present work investigated the underlying mechanism regarding the relationship between social support and different components of SWB in a sample of U.K. citizens during the COVID-19 pandemic. Results indicated that perceived social support prospectively predicted life satisfaction (i.e., cognitive SWB) through a full mediation of affect balance (i.e., affective SWB), and this predictive effect was moderated by people’s monthly household income. These findings contribute to the social support, SWB, and affect-as-information literature.

## Data Availability

The data are publicly accessible (https://osf.io/j2a8t/?view_only=6368555f2f494472bc77a2d841acc930).
